# Supplementary material for: Gemistocytic tumor cells programmed for glial scarring characterize T cell confinement in IDH-mutant astrocytoma
Source: Nat Commun. 2025 Jan 29;16:1156. doi: 10.1038/s41467-025-56441-5 (PMC11779865; doi:10.1038/s41467-025-56441-5)
Supplement: Supplementary file 1 — Supplementary Information [file 41467_2025_56441_MOESM1_ESM.pdf]

Supplementary figure 1

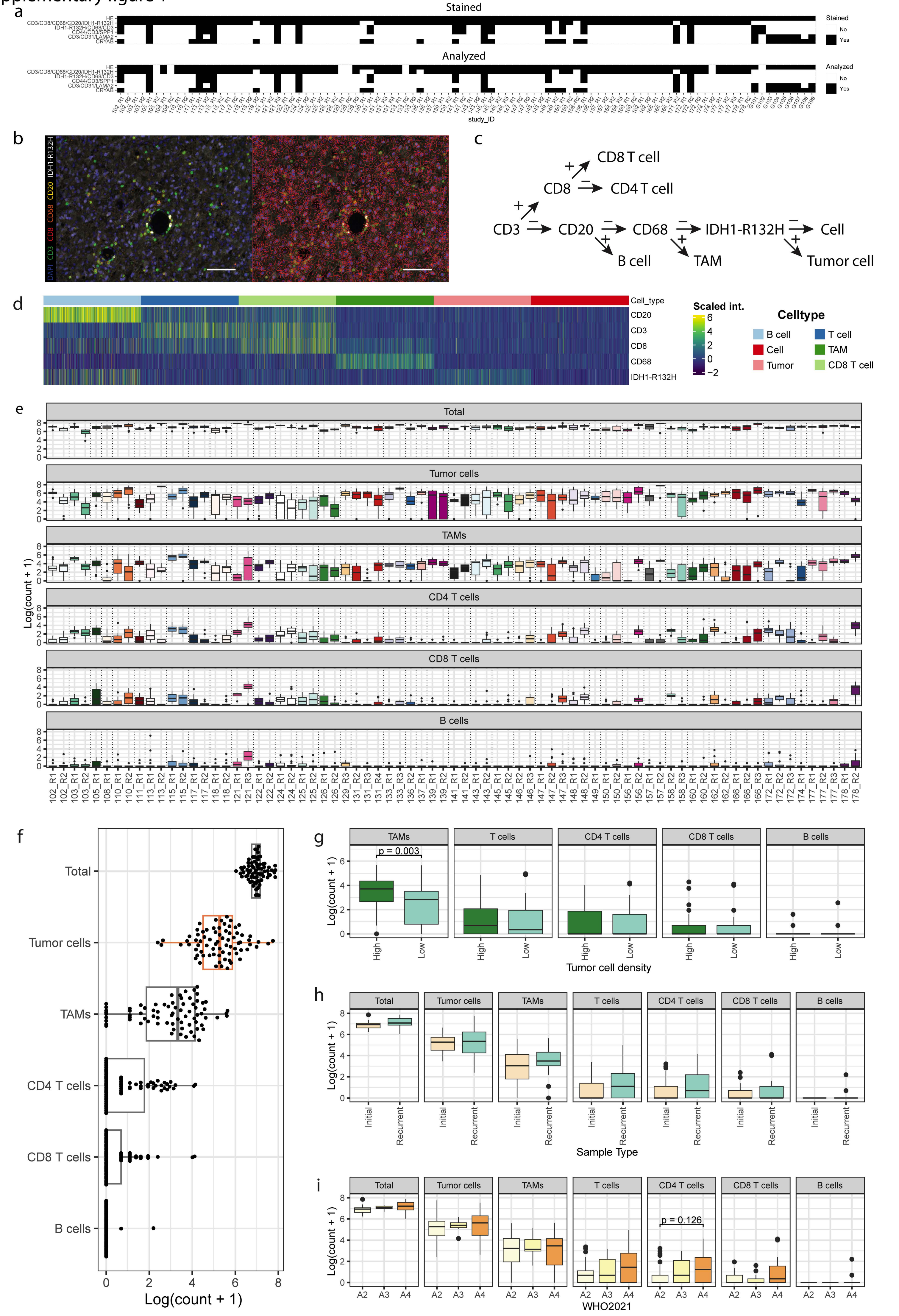

Supplementary figure 1: The immune cell compartment of IDHmt astrocytoma does not change over time or between tumor grades. **(a)** Overview of samples that have been stained and analyzed for multiplex IF panels. **(b)** Example of ROI with segmentation mask of nuclei and cytoplasm. **(c)** Cell type assignment strategy. **(d)** Log-transformed and scaled cell signal intensity values grouped by cell types of multiplex IF stainings (n = 75 samples, 2000 cells per cell type were sampled across all tumor samples). **(e)** Boxplots of all ROI cell counts for individual tumor samples. Patient samples are indicated with color. Resection number is indicated with R1-R4 (n = 1293 ROIs; n = 75 samples). **(f)** Boxplot of average cell count per tumor sample (n = 75 samples). **(g-i)** Comparison of average immune cell count between tumor-high and tumor-low regions (g; n = 67 tumor high, n = 54 tumor low)**,** initial- and recurrent tumor samples (h; n = 37 Initial, n = 33 Recurrent) or WHO grade (i; n = 42 A2, n = 11 A3, n = 22 A4). Wilcoxon rank sum test, two-sided, fdr corrected. TAM: tumor associated macrophage. Scale bars in b show 100 µm. Boxplots in e-i show the hinges at the first and third quartiles with the median as the center. The whiskers show min and max value until 1.5 times the interquartile range.

Supplementary figure 2

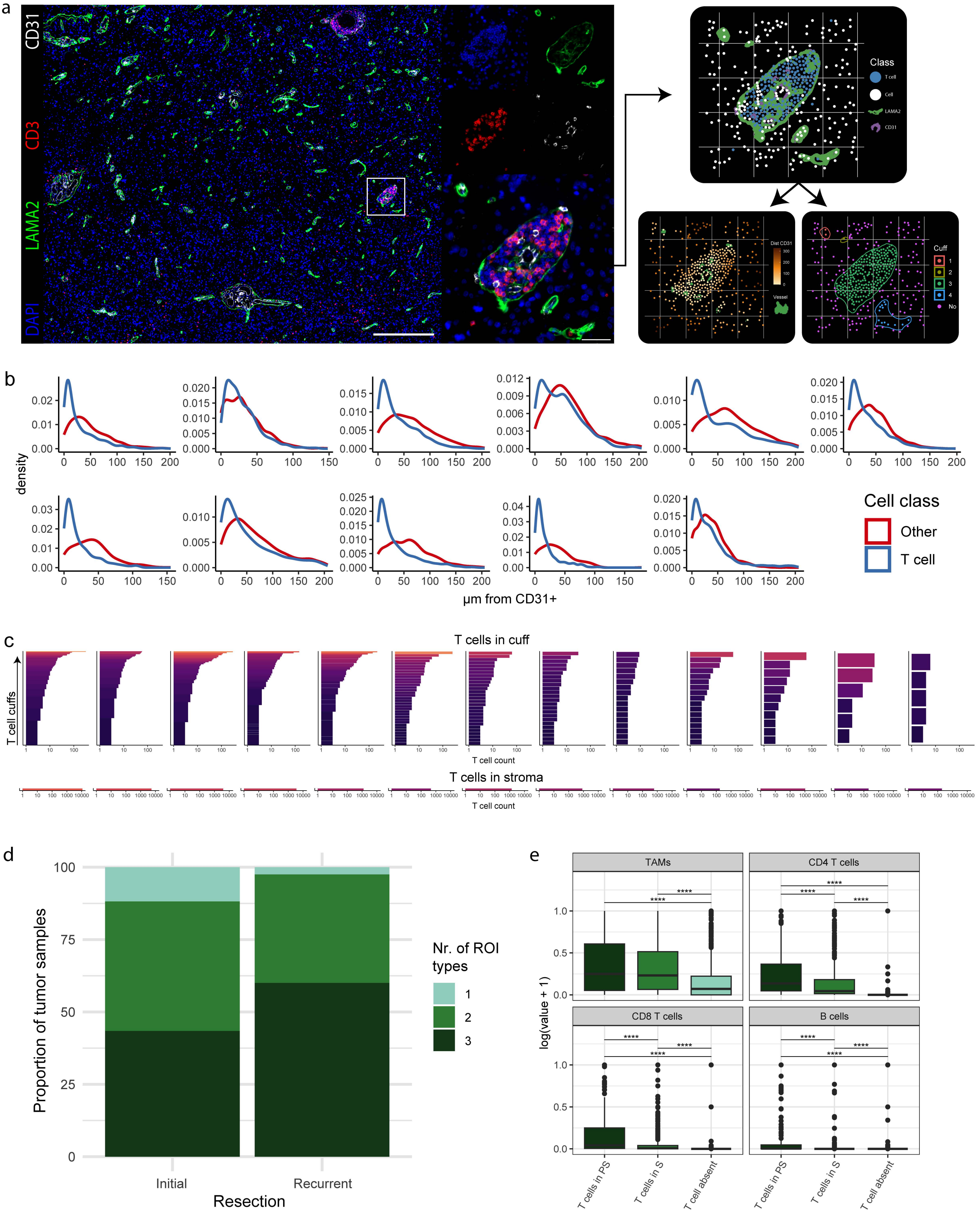

Supplementary figure 2: T cells are restricted to the perivascular space in IDHmt astrocytoma. **(a)** Example and workflow for multiplex IF stainings of perivascular T cell accumulation. **(b)** Density of distance to CD31+ vessels for T cells and other cells in all samples (n = 11). **(c)** Bar plots showing T cell quantities for individual T cell cuffs (top) and T cell quantities in tumor stroma (bottom) for individual samples (n = 13). **(d)** Bar plot of tumor sample fractions that contain one, two or all three of the T cell tissue phenotypes as shown in **Figure 1d** for initial and recurrent tumor samples (n = 37 Initial, n = 33 recurrent). **(e)** Cell counts per spatial tissue phenotype adjusted for tumor cell quantities (n = 634 T cells absent, n = 358 T cells in S, n = 183 T cells in PS). Wilcoxon rank sum test, two-sided, fdr corrected (p values from top to bottom per header: TAMs: 3.41×10^{17}, 6.44×10^{11}; CD4 T cells: 8.70×10^{105}, 6.65×10^{9}, 3.48×10^{136}; CD8 T cells: 3.87×10^{11}, 1.43×10^{77}, 3.40×10^{93}; B cells: 6.74×10^{19}, 1.13×10^{6}, 2.15×10^{47}). Dist: distance; S: stroma; PS: perivascular space; ROI: region of interest. Scale bar in a indicates 500 µm, Scale bar in insert indicates 50 µm. Boxplots in e show the hinges at the first and third quartiles with the median as the center. The whiskers show min and max value until 1.5 times the interquartile range.

Supplementary figure 3

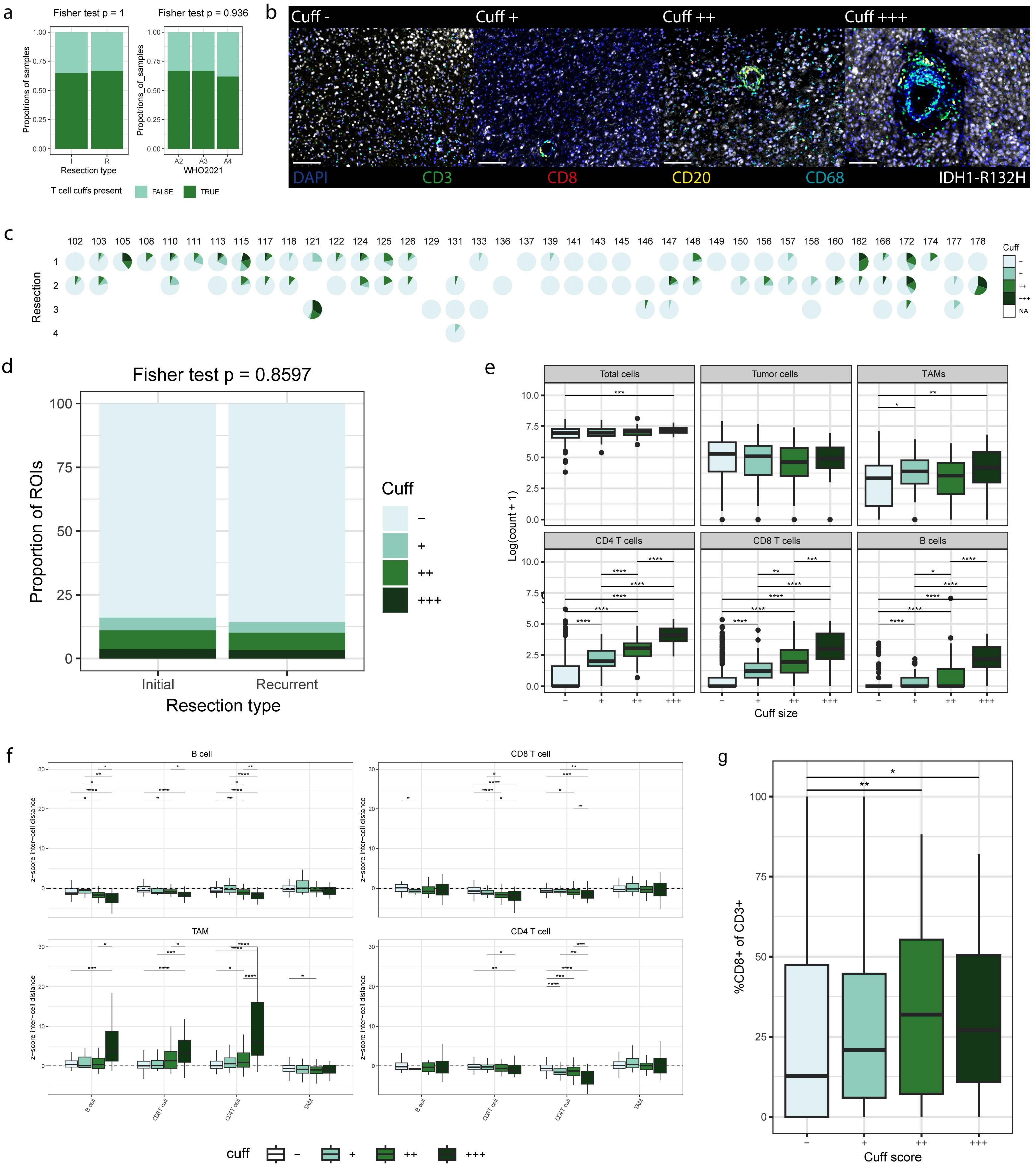

Supplementary figure 3: T cell cuff presence and size are reliably associated with an accumulation of lymphocytes but not tumor cells or TAMs. **(a)** Bar plots depicting proportions of tumor samples containing T cell cuffs split by resection type or WHO grade (n = 37 Initial, n = 33 Recurrent; n = 39 A2, n = 9 A3, n = 21 A4). **(b)** Examples for T cell cuff ROI bins. **(c)** Presence of perivascular T cell cuffs throughout the patient cohort. **(d)** Proportions of ROIs containing T cell cuffs as shown in (b) in initial or recurrent tumor samples (n = 591 Initial, n = 545 Recurrent). **(e)** Cell counts from all ROIs grouped by cuff score (n = 1026 -, n = 56 +, n = 84 ++, n = 44 +++). Wilcoxon rank sum test, two-sided, fdr corrected (p values from top to bottom per header: Total cells: 0.0003; TAMs: 0.0019, 0.0144; CD4 T cells: 3.70×10^{-10}, 7.33×10^{-6}, 1.47×10^{-13}, 5.78×10^{-29}, 1.93×10^{-36}, 2.41×10^{-15}; CD8 T cells: 0.0009, 0.0019, 2.00×10^{-7}, 1.07×10^{-26}, 3.30×10^{-32}, 4.35×10^{-14}; B cells: 7.47×10^{-10}, 0.0189, 4.48×10^{-14}, 7.85×10^{-62}, 1.90×10^{-21}, 8.45×10^{-5}). **(f)** Boxplot of z-scores for inter-cell distances between two cell types (between the cell types indicated in the plot title and the x-axis names) for all ROIs grouped by cuff score (n is shown in **Supplementary Data 16**). Wilcoxon rank sum test, two-sided, fdr corrected (p values from top to bottom, left to right, per header: B cell: 0.012, 0.0003, 0.02, 3.08×10^{-6}, 0.012, 0.0177, 8.80×10^{-6}, 0.0455, 0.002, 4.68×10^{-5}, 0.0178, 1.09×10^{-7}, 0.0083; CD8 T cell: 0.033, 0.0427, 8.80×10^{-6}, 7.64×10^{-7}, 0.033, 0.0083, 0.0001, 0.0115, 0.032; TAM: 0.0115, 0.0002, 0.0205, 0.0001, 1.34×10^{-6}, 1.69×10^{-7}, 4.16×10^{-12}, 0.0298, 2.14×10^{-5}, 0.0441; CD4 T cell: 0.0427, 0.0026, 0.0008, 0.0048, 9.31×10^{-11}, 0.0002, 8.80×10^{-6}). **(g)** ROI CD8+ T cell fractions of total T cell counts separated for cuff score (n = 540 -, n = 56 +, n = 84 ++, n = 44 +++). Wilcoxon rank sum test, two-sided, fdr corrected (p values from top to bottom: 0.045, 0.0009). TAM: tumor associated macrophage. Boxplots in **e**-**g** show the hinges at the first and third quartiles with the median as the center. The whiskers show min and max value until 1.5 times the interquartile range.

Supplementary figure 4

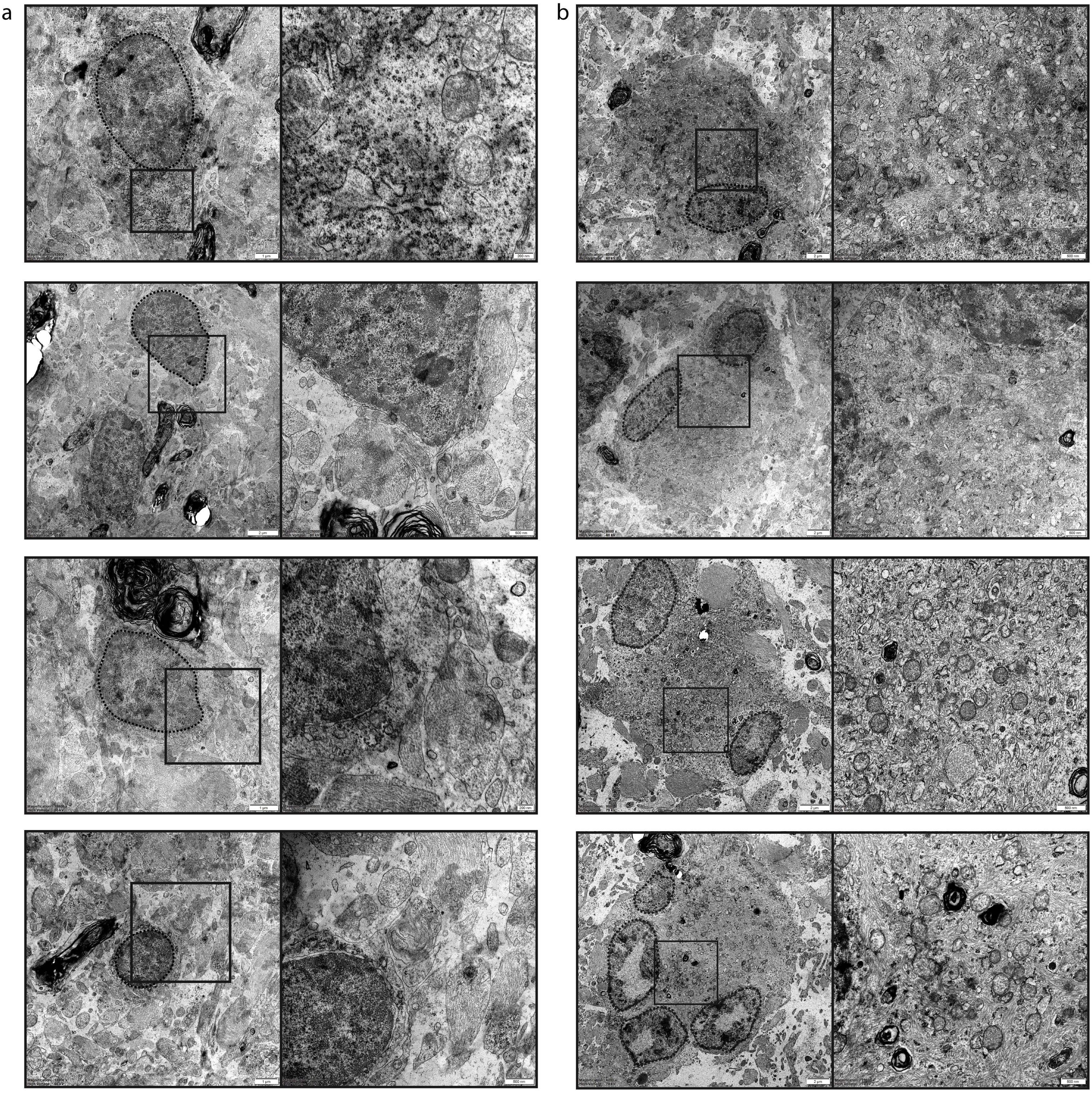

Supplementary figure 4: EM recordings of non-gemistocytic **(a)** and gemistocytic **(b)** cells. Inserts in images (left) indicate magnified areas (right). Dashed lines indicate nuclei.

Supplementary figure 5

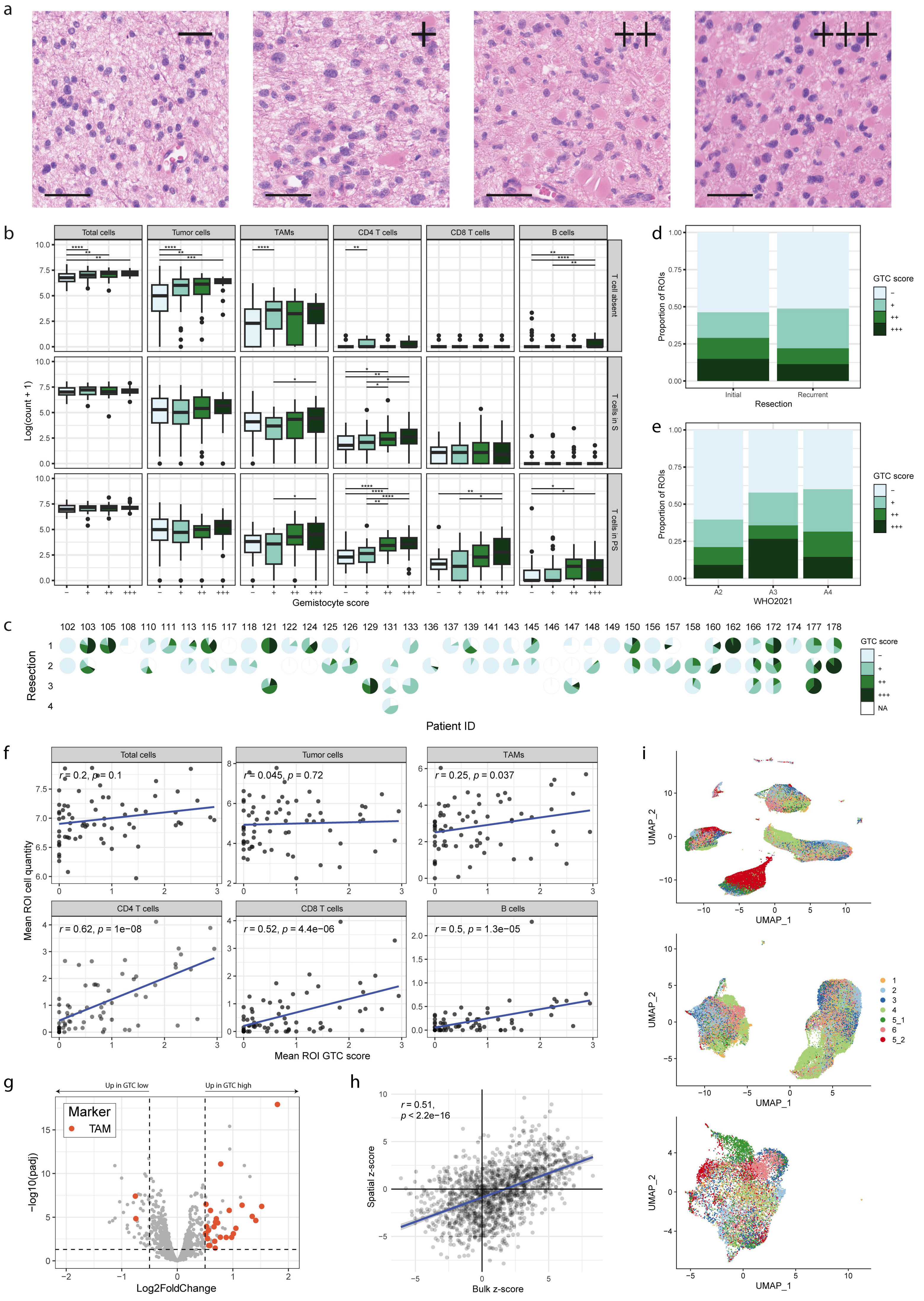

Supplementary figure 5: GTCs reliably associate with immune cell signatures across analysis platforms. **(a)** Representative images of ROI GTC quantity scores for H&E stainings. **(b)** Cell quantities split by GTC score and spatial tissue phenotype (n is shown **Supplementary Data 17**). Wilcoxon rank sum test, two-sided, fdr corrected (p values displayed in panels from left to right, top to bottom: panel 1: 8.28×10^{-5}, 0.0052, 0.0032; panel 2: 1.08×10^{-5}, 0.0113, 0.0008; panel 3: 8.28×10^{-5}; panel 4: 0.0003; panel 6: 0.0030, 8.28×10^{-5}, 0.0114; panel 9: 0.0147; panel 10: 0.002, 0.0003, 0.002, 0.012; panel 15: 0.006; panel 16: 8.28×10^{-5}, 4.51×10^{-6}, 0.0003, 0.0052; panel 17: 0.0052, 0.0389; panel 18: 0.0147, 0.0147). **(c)** Distribution of GTC scores across the patient cohort according to bins illustrated in **a**. **(d,e)** Proportion of ROI GTC scores split by primary and recurrent resections (d; n = 469 Initial, n = 441 Recurrent) and WHO grade (e; n = 518 A2, n = 154 A3, n = 270 A4). **(f)** Correlation of average sample cell quantities with average sample GTC scores. Pearson's r, correlation t-test (n = 57). **(g)** Volcano plot of the DE test between GTC-low (n = 47) and high (n = 21) ROIs from the NanoString GeoMx DSP spatial transcriptomics data. Wald test, two-sided, fdr corrected. Lines indicate fdr-adjusted p-value and log2FoldChange cutoffs. **(h)** Correlation between z-scores from the GTC-high versus low DE results for bulk and spatial RNA sequencing. Pearson's r, correlation t-test. **(i)** UMAP representations of snRNA-seq data of all cells (top, n = 65,129), tumor cells (middle; n = 42,011) and TAMs (bottom; n = 10775). The colors represent tumor samples. S: stroma; PS: perivascular space; GTC: gemistocytic tumor cell; TAM: tumor associated macrophage; ROI: region of interest. Scale bars in a indicate 50 µm. Figure h shows error bounds of ± SEM. Boxplots in b show the hinges at the first and third quartiles with the median as the center. The whiskers show min and max value until 1.5 times the interquartile range.

Supplementary figure 6

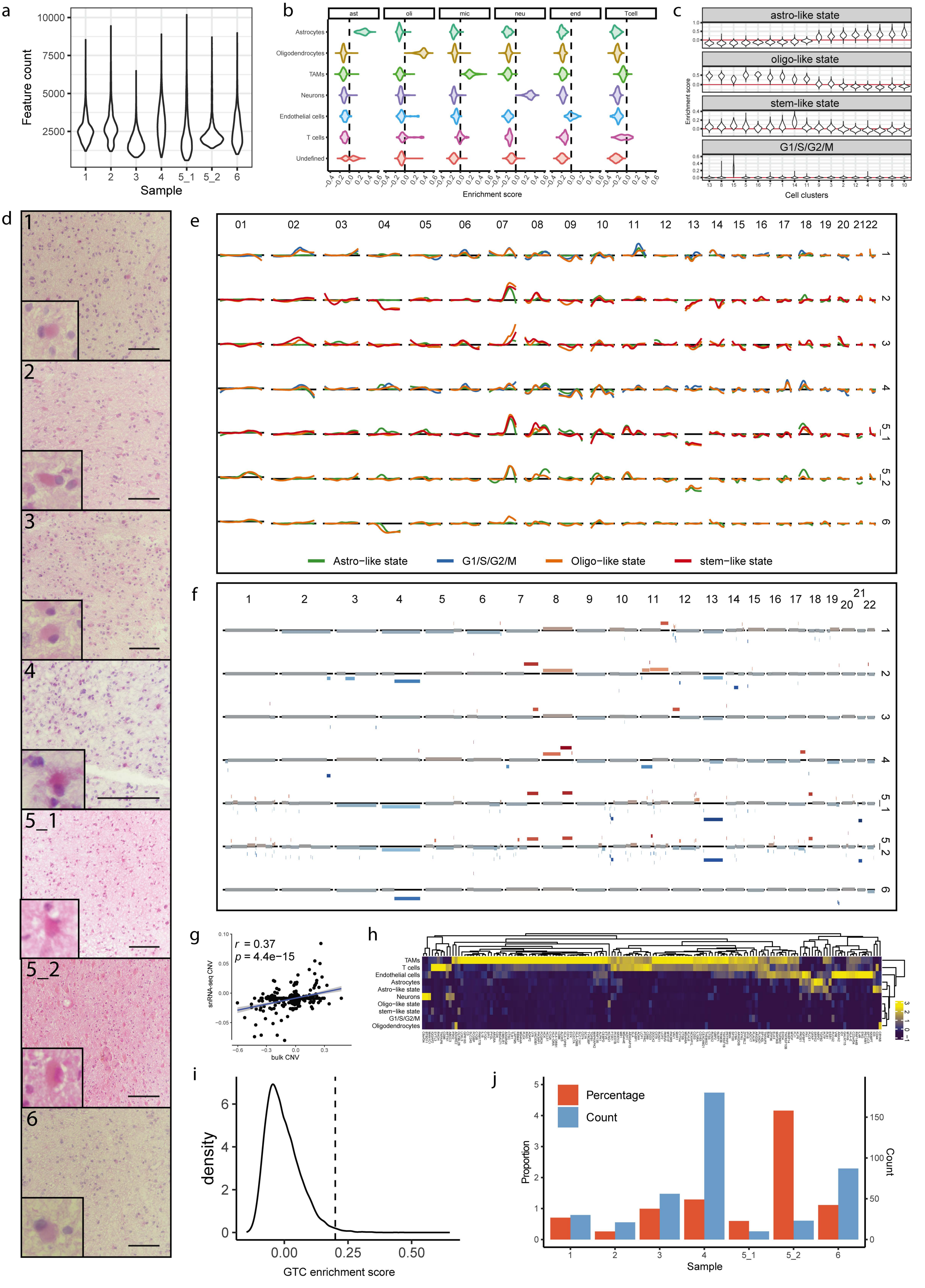

Supplementary figure 6: Combined bulk and snRNA-seq analysis identifies the GTCs transcriptomic profile. **(a)** Feature counts for seven snRNA-seq samples. **(b)** Marker gene enrichment scores for cell types from the integrated snRNA-seq data object. **(c)** Cluster-specific enrichment scores of tumor cell transcriptional states for the integrated snRNA-seq tumor cell object from **Figure 3b**. **(d)** H&E stainings for all snRNA-seq samples showing presence of gemistocytic cells. **(e)** Inferred copy number variation (CNV) estimates of transcriptional tumor cell states from snRNA-seq data of seven IDHmt astrocytoma samples. **(f)** CNV estimates from bulk methylation profiles of matching samples from e. **(g)** Correlation of CNV profiles from e and f. Pearson's r, correlation t-test. **(h)** Heatmap depicting average expression of GTC-high bulk DE genes in snRNA-seq cell populations from b. **(i)** Density plot of the GTC enrichment score in the snRNA-seq tumor cell populations. The vertical line represents the cutoff value for GTCs. **(j)** GTC quantity in count and percentage of all tumor cells per sample. Ast: astrocyte; oli: oligodendrocyte; mic: microglia; neu: neuron; end: endothelial cells; TAM: tumor associated macrophage; GTC: gemistocytic tumor cell; CNV: copy number variation. Scale bars in d show 100 µm.

Supplementary figure 7

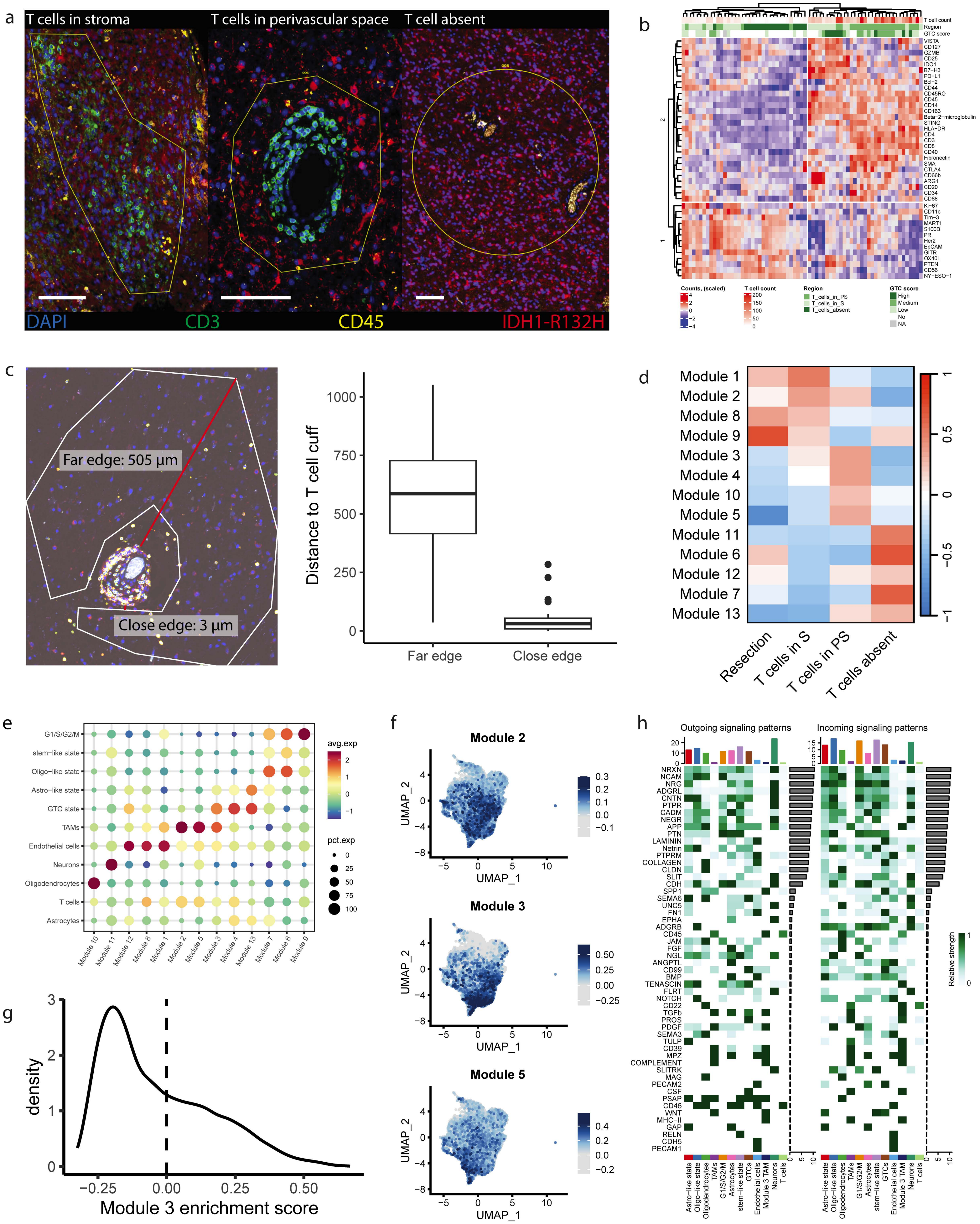

Supplementary figure 7: Spatial analyses of RNA and protein expression verify association between GTCs and reactive TAMs. **(a)** Representative ROIs from NanoString GeoMx DSP spatial proteomics data. In this dataset, ROIs for T cells in the perivascular space include T cell cuffs. **(b)** Heatmap showing all protein detections from the NanoString GeoMx DSP spatial proteomics data (n = 69). **(c)** Example ROI (left) and boxplot (right) showing close edge and far edge distance to the nearest T cell cuff of ROIs annotated as T cells in perivascular space in the NanoString GeoMx DSP spatial transcriptomics data (n = 25). **(d)** Correlation matrix of ROI annotations with all gene modules as identified by WGCNA in the NanoString GeoMx DSP spatial transcriptomics data. Pearson's r. **(e)** Enrichment scores for the gene modules from d in the snRNA-seq cell populations. **(f)** Enrichment scores of gene modules 2, 3 and 5 in the TAM snRNA-seq population. **(g)** Density plot of module 3 enrichment score for all TAMs. The vertical line represents the cutoff value for module 3 positive TAMs. **(h)** Heatmap showing relative strength of outgoing and incoming signaling patterns for significant pathways in every cell population from the integrated snRNA-seq dataset. S: stroma; PS: perivascular space; TAM: tumor associated macrophage; GTC: gemistocytic tumor cell; ROI: region of interest. Scale bars in a represent 100 µm. The Boxplots in c show the hinges at the first and third quartiles with the median as the center. The whiskers show min and max value until 1.5 times the interquartile range.

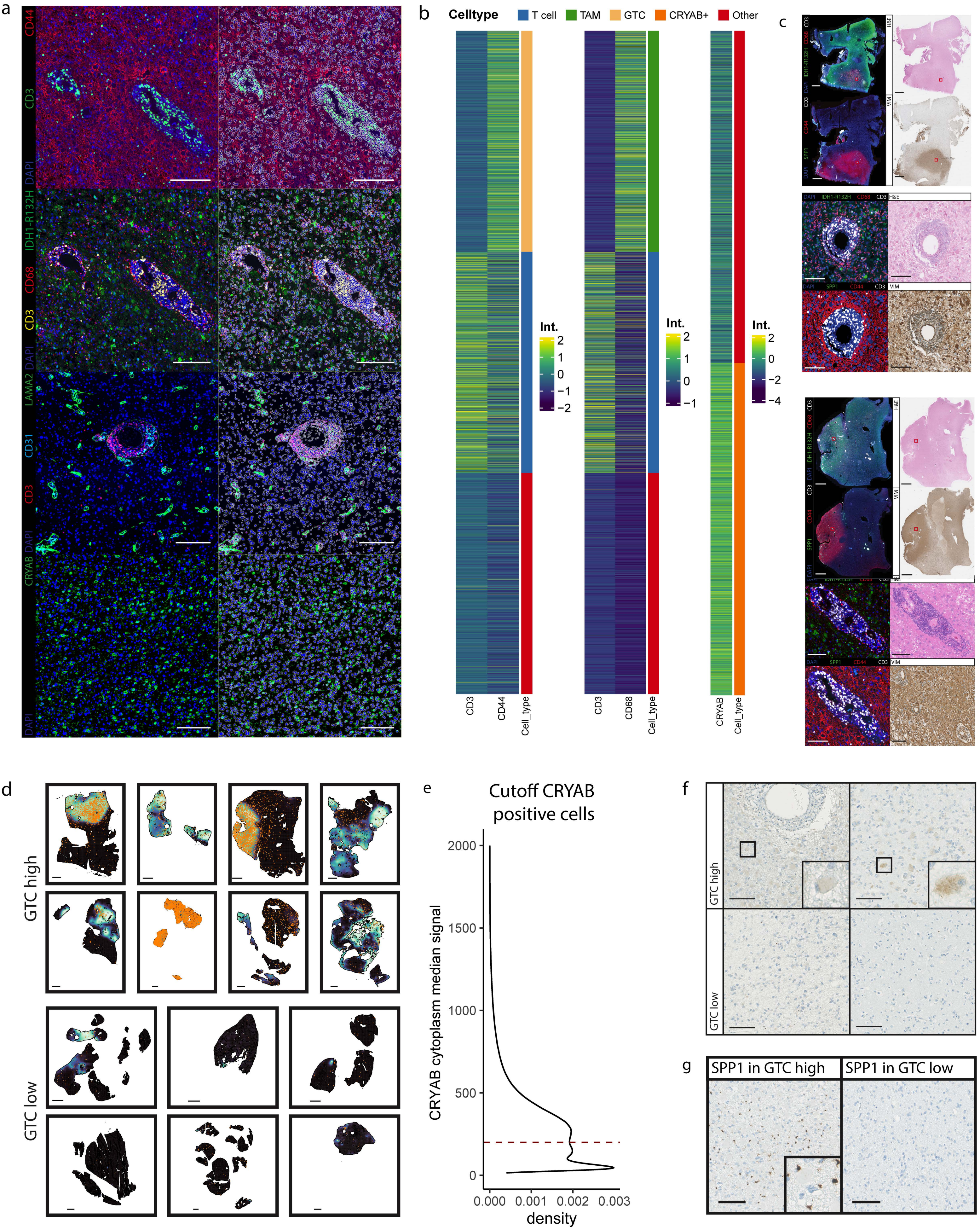

a
CD44
CD3
DAPI
IDH1-R132H
CD68
CD3
DAPI
LAMA2
CD31
CD3
DAPI
CRYAB
DAPI
b
Celltype
T cell
TAM
GTC
CRYAB+
Other
Int.
CD3
CD44
Cell_type
CD3
CD68
Cell_type
CRYAB
Cell_type
c
H&E
VIM
d
GTC high
GTC low
e
Cutoff CRYAB positive cells
CRYAB cytoplasm median signal
density
f
GTC high
GTC low
g
SPP1 in GTC high
SPP1 in GTC low

Supplementary figure 8: Gemistocytic tumor cells spatially associate with leukocyte accumulation. **(a)** Example images of validation multiplex stainings with nucleus and cytoplasm segmentation masks. **(b)** Heatmaps of log-transformed and scaled signal intensity values of multiplex IF stainings grouped by cell types (from left to right, n = 9, n = 9, n = 30; 2000 cells per cell type were sampled across all tumor samples). **(c)** Example whole slide images of two gemistocyte high samples. Images show HE, VIM and multiplex IF stainings for CD44, CD3, CD68, SPP1, and IDH1-R132H on consecutive tissue sections. Inserts show presence of perivascular T cell cuffs in CD44 high regions. **(d)** Adjusted spatial CD44 kernel density plots of whole slide image scans from GTC-high and low samples overlaid with T cell locations (orange). **(e)** Density plot showing CRYAB intensity for all samples. The red line indicates the cutoff for CRYAB positive cells. **(f)** Representative images of IHC stainings for IL-1β in GTC-high and GTC-low regions. **(g)** Representative images of IHC stainings for SPP1 in GTC-high and low tumor samples. GTC: gemistocytic tumor cell. Scale bars in **a** indicate 200 µm. Scale bars in **c** and **d** indicate 2 mm. Scale bars in insets of **c**, **f** and **g** indicate 100 µm.
