# Supplementary material for: Gemistocytic tumor cells programmed for glial scarring characterize T cell confinement in IDH-mutant astrocytoma
Source: Nat Commun. 2025 Jan 29;16:1156. doi: 10.1038/s41467-025-56441-5 (PMC11779865; doi:10.1038/s41467-025-56441-5)
Supplement: Supplementary file 2 — Description of Additional Supplementary Files [file 41467_2025_56441_MOESM2_ESM.pdf]

Description of Additional Supplementary Files

Supplementary Data 1: Sample information multiplex immunofluorescence VECTRA data.

Supplementary Data 2: Sample information bulk RNA sequencing data.

Supplementary Data 3: Sample information NanoString GeoMx spatial transcriptomics data.

Supplementary Data 4: Sample information single nucleus RNA-sequencing data.

Supplementary Data 5: Sample and ROI information NanoString GeoMx protein data.

Supplementary Data 6: Gemistocytic tumor cell and reactive microglia markers.

Supplementary Data 7: Sample information whole slide IF validation stainings.

Supplementary Data 8: Raw protein count data.

Supplementary Data 9: Cell counts and distance metrics multiplex IF VECTRA images.

Supplementary Data 10: Markers cell type identification single nucleus RNA-sequencing.

Supplementary Data 11: Reactive astrocyte markers reported in literature.

Supplementary Data 12: Markers Seurat clusters related to Figure 3b.

Supplementary Data 13: Bulk IPA pathway enrichment.

Supplementary Data 14: WGCNA gene modules.

Supplementary Data 15: Nearest neighbor tests related to Figure 1i

Supplementary Data 16: nearest neighbor tests related to Supplementary figure 3f

Supplementary Data 17: Statistical test results related to figure 5b
