# Supplementary material for: Gemistocytic tumor cells programmed for glial scarring characterize T cell confinement in IDH-mutant astrocytoma
Source: Nat Commun. 2025 Jan 29;16:1156. doi: 10.1038/s41467-025-56441-5 (PMC11779865; doi:10.1038/s41467-025-56441-5)
Supplement: Supplementary file 4 — Reporting Summary [file 41467_2025_56441_MOESM4_ESM.pdf]

nature portfolio

Corresponding author(s): Levi van Hijfte

Last updated by author(s): Dec 18, 2024

Reporting Summary

Nature Portfolio wishes to improve the reproducibility of the work that we publish. This form provides structure for consistency and transparency in reporting. For further information on Nature Portfolio policies, see our Editorial Policies and the Editorial Policy Checklist.

Statistics

For all statistical analyses, confirm that the following items are present in the figure legend, table legend, main text, or Methods section.

n/a	Confirmed	
☐	☒	The exact sample size (*n*) for each experimental group/condition, given as a discrete number and unit of measurement
☐	☒	A statement on whether measurements were taken from distinct samples or whether the same sample was measured repeatedly
☐	☒	The statistical test(s) used AND whether they are one- or two-sided<br>*Only common tests should be described solely by name; describe more complex techniques in the Methods section.*
☐	☒	A description of all covariates tested
☐	☒	A description of any assumptions or corrections, such as tests of normality and adjustment for multiple comparisons
☐	☒	A full description of the statistical parameters including central tendency (e.g. means) or other basic estimates (e.g. regression coefficient) AND variation (e.g. standard deviation) or associated estimates of uncertainty (e.g. confidence intervals)
☐	☒	For null hypothesis testing, the test statistic (e.g. *F*, *t*, *r*) with confidence intervals, effect sizes, degrees of freedom and *P* value noted<br>*Give P values as exact values whenever suitable.*
☒	☐	For Bayesian analysis, information on the choice of priors and Markov chain Monte Carlo settings
☐	☒	For hierarchical and complex designs, identification of the appropriate level for tests and full reporting of outcomes
☐	☒	Estimates of effect sizes (e.g. Cohen's *d*, Pearson's *r*), indicating how they were calculated

Our web collection on statistics for biologists contains articles on many of the points above.

Software and code

Policy information about availability of computer code

Data collection: The inForm (2.4.8) software was used to collect and process ROI images from multiplex IF stainings that were imaged with the VECTRA 3.0 system. ZEN (3.9) was used to collect whole slide image scans from multiplex immunofluorescence stainings from the LSM 700 or Axioscan systems. NDP.scan (2.5.90) software was used to acquire HE and IHC staining image scans with the NanoZoomer 2.0HT system. The NanoString GeoMx Digital Spatial Profiler (Analysis Suite version 3.1.0.222) was used to acquire NanoString GeoMx DSP transcriptomics and proteomics data.

Data analysis: TME-analyzer (2.3; Balcioglu et al.; npj Imaging) was used for ROI image analysis. QuPath (0.5.0) was used for whole slide image analysis. Ingenuity Pathway Analysis (Qiagen; 24.0) was used for pathway enrichment analysis of bulk RNA sequencing data. R (4.3.0) was used for further image analysis and visualization. The following R packages were used: WGCNA (1.72-5); fastcluster (1.2.3); dynamicTreeCut (1.63-1); infercnv (1.16.0); ggbeeswarm (0.7.2); ggpubr (0.6.0); DESeq2 (1.40.1); SummarizedExperiment (1.30.2); Biobase (2.60.0); MatrixGenerics (1.12.3); matrixStats (1.0.0); GenomicRanges (1.52.0); GenomeInfoDb (1.36.0); IRanges (2.34.0); S4Vectors (0.38.1); BiocGenerics (0.46.0); readxl (1.4.3); Seurat (5.1.0); SeuratObject (5.0.2); sp (2.1-2); rstatix (0.7.2); viridis (0.6.4); viridisLite (0.4.2); ggrepel (0.9.4); ggridges (0.5.4); corrplot (0.92); circlize (0.4.15); patchwork (1.3.0); lubridate (1.9.3); forcats (1.0.0); stringr (1.5.1); dplyr (1.1.2); purrr (1.0.1); readr (2.1.4); tidyr (1.3.0); tibble (3.2.1); ggplot2 (3.5.1); tidyverse (2.0.0); sctransform (0.4.1); RColorBrewer (1.1-3); ComplexHeatmap (2.16.0); VennDiagram (1.7.3); futile.logger (1.4.3); sf (1.0-17); spatstat (3.1-1); stars (0.6-6)

For manuscripts utilizing custom algorithms or software that are central to the research but not yet described in published literature, software must be made available to editors and reviewers. We strongly encourage code deposition in a community repository (e.g. GitHub). See the Nature Portfolio guidelines for submitting code & software for further information.

Data

Policy information about availability of data

All manuscripts must include a data availability statement. This statement should provide the following information, where applicable:

- Accession codes, unique identifiers, or web links for publicly available datasets
- A description of any restrictions on data availability
- For clinical datasets or third party data, please ensure that the statement adheres to our policy

VECTRA ROI images are available on Zenodo and processed count and cell-cell distance values can be found in supplementary documents (DOI: 10.5281/zenodo.13911692; Table S9). Processed count data from 10x single nucleus RNA sequencing experiments are available on Zenodo (DOI: 10.5281/zenodo.10435521; DOI). NanoString GeoMx spatial transcriptomics data is available on Zenodo and sample annotations are provided in supplementary documents (DOI: 10.5281/zenodo.13911761; Table S1)33. NanoString GeoMx spatial proteomics data can be found in the supplementary documents, ROI images are available at Zenodo (Table S6; DOI: 10.5281/zenodo.13918032). Whole-slide scans of multipex IF stainings and corresponding GeoJSON and measurement files are available on Zenodo (CRYAB/HE: 10.5281/zenodo.13918123; CD31, CD3, LAMA2: 10.5281/zenodo.13918377; CD44, CD3, SPP1, TNC and CD68, IDH1-R132H, CD3, CD31: 10.5281/zenodo.13911899; Table S7) Bulk RNA and methylation data are under embargo and can be found upon publication27.

Research involving human participants, their data, or biological material

Policy information about studies with human participants or human data. See also policy information about sex, gender (identity/presentation), and sexual orientation and race, ethnicity and racism.

Reporting on sex and gender	Sex is reported for all samples. No analysis were done with regard to sex.
Reporting on race, ethnicity, or other socially relevant groupings	NA
Population characteristics	All relevant patient characteristics were reported. Analyses were not corrected for these characteristics.
Recruitment	For the GLASS-NL study, patients were selected that had undergone more than one resection. Further relevant details will be described here https://doi.org/10.1101/2024.03.05.583306.
Ethics oversight	Ethical board of the Erasmus MC (MEC-2019-0288, Rotterdam, The Netherlands)

Note that full information on the approval of the study protocol must also be provided in the manuscript.

Field-specific reporting

Please select the one below that is the best fit for your research. If you are not sure, read the appropriate sections before making your selection.

☒ Life sciences ☐ Behavioural & social sciences ☐ Ecological, evolutionary & environmental sciences

For a reference copy of the document with all sections, see nature.com/documents/nr-reporting-summary-flat.pdf

Life sciences study design

All studies must disclose on these points even when the disclosure is negative.

Sample size	No statisctical methods were used to determine sample size.
Data exclusions	Imaging data was excluded if staining quality was poor or if imaging quality was poor. Samples were excluded if they had no IDH1-R132H mutation. For VECTRA ROI analysis, samples that had fewer than 3 ROIs after QC were excluded. In single nucleus RNA sequencing data, cells were excluded if they were estimated to be doublets, if they had an insufficient UMI count or if they could not be assigned to a known cell population. For NanoString GeoMx DSP data, ROIs were excluded that contained low RNA or protein detection rates. Details on data exclusion can be found in the manuscript.
Replication	NA
Randomization	NA
Blinding	Investigators were blinded for clinical and sample information throughout our analysis.

Reporting for specific materials, systems and methods

We require information from authors about some types of materials, experimental systems and methods used in many studies. Here, indicate whether each material, system or method listed is relevant to your study. If you are not sure if a list item applies to your research, read the appropriate section before selecting a response.

Materials & experimental systems

n/a	Involved in the study
☐	☒ Antibodies
☒	☐ Eukaryotic cell lines
☒	☐ Palaeontology and archaeology
☒	☐ Animals and other organisms
☒	☐ Clinical data
☒	☐ Dual use research of concern
☒	☐ Plants

Methods

n/a	Involved in the study
☒	☐ ChIP-seq
☒	☐ Flow cytometry
☒	☐ MRI-based neuroimaging

Antibodies

Antibodies used	anti-VIM (Cell Marque, #347M-10); anti-IL1β (Abcam, #ab9722); anti-SPP1 (Santa Cruz, #D2723); anti-CD56 (Cell Marque, #MRQ-42); anti-CD3 (Sigma-Aldrich, #SAB5500058); anti-CD20 (Perklin Elmer, #OP4LY2001KT); anti-CD8 (Cell Marque, #108M-96); anti-CD68 (Cell marque, #168M-96) , anti-IDH1-R132H (Dianova, #DIA-H09); anti-CD3 (Ventana, #2GV6); anti-LAMA2 (Sigma Aldrich, #5H2); anti-CD31 (Cell Marque, #JC70); anti-CD68 (Ventana, #KP1); anti-CD44 (Novusbio, #8E2F3); anti-SPP1 (Santa Cruz, #D2723); anti-CRYAB (Abcam, #1B6-13g4)
Validation	All antibodies are commercially available and the relevant information is available at manufacturer website

Plants

Seed stocks	NA
Novel plant genotypes	NA
Authentication	NA
